# International *Clostridium difficile* animal strain collection and large diversity of animal associated strains

**DOI:** 10.1186/1471-2180-14-173

**Published:** 2014-06-28

**Authors:** Sandra Janezic, Valerija Zidaric, Bart Pardon, Alexander Indra, Branko Kokotovic, Jose Luis Blanco, Christian Seyboldt, Cristina Rodriguez Diaz, Ian R Poxton, Vincent Perreten, Ilenia Drigo, Alena Jiraskova, Matjaz Ocepek, J Scott Weese, J Glenn Songer, Mark H Wilcox, Maja Rupnik

**Affiliations:** 1 National Laboratory for Health, Environment and Food, Maribor, Slovenia; 2 Department of Large Animal Internal Medicine, Faculty of Veterinary Medicine, Ghent University, Merelbeke, Belgium; 3 Austrian Agency for Health and Food Safety (AGES), Vienna, Austria; 4 Technical University of Denmark, National Veterinary Institute, Copenhagen, Denmark; 5 Complutense University, Madrid, Spain; 6 Friedrich-Loeffler-Institut, Federal Research Institute for Animal Health, Institute of Bacterial Infections and Zoonoses, Jena, Germany; 7 University of Liege, Faculty of Veterinary Medicine, Liege, Belgium; 8 University of Edinburgh, Edinburgh, UK; 9 University of Bern, Institute of Veterinary Bacteriology, Bern, Switzerland; 10 IZSVe, Treviso, Italy; 11 Charles University in Prague, 1st Faculty of Medicine, Prague, Czech Republic; 12 University of Ljubljana, Veterinary Faculty, Ljubljana, Slovenia; 13 University of Guelph, Ontario Veterinary College, Ontario, Canada; 14 Department of Veterinary Science and Microbiology, University of Arizona, Tucson, USA; 15 Department of Medical Microbiology, Leeds Teaching Hospitals, Leeds, UK; 16 University of Maribor, Medical Faculty, Maribor, Slovenia; 17 Centre of Excellence for Integrated Approaches in Chemistry and Biology of Proteins, Ljubljana, Slovenia

**Keywords:** *Clostridium difficile*, Animals, Ribotyping, Geographic distribution, Strain collection

## Abstract

**Background:**

*Clostridium difficile* is an important cause of intestinal infections in some animal species and animals might be a reservoir for community associated human infections. Here we describe a collection of animal associated *C. difficile* strains from 12 countries based on inclusion criteria of one strain (PCR ribotype) per animal species per laboratory.

**Results:**

Altogether 112 isolates were collected and distributed into 38 PCR ribotypes with agarose based approach and 50 PCR ribotypes with sequencer based approach. Four PCR ribotypes were most prevalent in terms of number of isolates as well as in terms of number of different host species: 078 (14.3% of isolates; 4 hosts), 014/020 (11.6%; 8 hosts); 002 (5.4%; 4 hosts) and 012 (5.4%; 5 hosts). Two animal hosts were best represented; cattle with 31 isolates (20 PCR ribotypes; 7 countries) and pigs with 31 isolates (16 PCR ribotypes; 10 countries).

**Conclusions:**

This results show that although PCR ribotype 078 is often reported as the major animal *C. difficile* type, especially in pigs, the variability of strains in pigs and other animal hosts is substantial. Most common human PCR ribotypes (014/020 and 002) are also among most prevalent animal associated *C. difficile* strains worldwide. The widespread dissemination of toxigenic *C. difficile* and the considerable overlap in strain distribution between species furthers concerns about interspecies, including zoonotic, transmission of this critically important pathogen.

## Background

*Clostridium difficile*, an anaerobic sporogenic bacterium, is recognized as the major pathogen in healthcare associated intestinal infections in humans and also as an important animal pathogen. In addition to the potential for serious (including fatal) infections in animals, are companion and food animals considered as an important potential source for human community-acquired infections [1-4]. This indicates the importance of preventive measures targeting animals and food [5]. Several studies have looked at similarity between strains isolated from humans and animals [1,6-10], but typically focusing on limited number of different species and restricted to a narrow geographic region.

PCR ribotyping is currently the method of choice for differentiation of *C. difficile* strains. In humans more than 300 PCR ribotypes are recognized while the number of reported animal associated PCR ribotypes is much lower [1,8,11]. It could be expected that variety of animal associated *C. difficile* will increase with the increased number of typed animal isolates. To date piglets and pig farms are hosts and environments where *C. difficile* has been most extensively studied [3,12-18]. For this reservoir the modes of transmission and potential association with human infections are also best understood [14,16].

*C. difficile* strains can also be differentiated into toxinotypes according to the differences in the toxin A and toxin B encoding region (PaLoc) [19]. Toxinotypes V and XI are particularly often isolated from animals [19], but the reason for this association is not known. Many published studies report *C. difficile* PCR ribotypes, but not many do report the toxinotypes [9,13].

The aim of this study was to collect *C. difficile* isolates from different countries and different animals and to compare them with classical agarose gel-based and sequencer-based PCR ribotyping and determine the toxinotypes.

## Results

Altogether 112 *C. difficile* isolates from 13 animal species were obtained from 14 laboratories from 12 different countries (Austria, Belgium, Canada, Czech Republic, Denmark, Germany, Italy, Scotland, Slovenia, Spain, Switzerland and USA) (Table 1, Additional file 1). Inclusion criteria were one strain (PCR ribotype) per animal species per laboratory. Each participating country contributed 1 to 24 isolates (Additional file 1). Strains were isolated between 1998 and 2012. Only five isolates were from 1998 to 2002, with the majority of isolates (n = 34) from 2011.

**Table 1:** **PCR ribotypes and toxinotypes represented in the international collection of animal *C. difficile *** **strains**

			**Number of strains/different countries per animal species**						**In total**	
**PCR ribotype**	**WEBRIBO type**	Toxinotype*	**Cattle**	**Horse**	**Pig**	**Poultry**	**Cats and dogs**	Others**	**Nr. of strains (%)**	**Nr. of countries**
078	078, 078/4	V/Btb+	5/4	2/2	8/7			1/1	16 (14,3)	9
014/020	014/0, 014/5, 020, 449,659	0	3/3	1/1	2/1	1/1	4/2	2/2	13 (11,6)	5
002	203, 209	0	2/2		2/2		1/1	1/1	6 (5,4)	5
012	012	0, XIX	2/2		1/1	1/1	1/1	1/1	6 (5,4)	4
010	010	tox-	1/1			1/1	2/2	1/1	5 (4,5)	4
033	033	XIa, XIb/Btb+	3/3	1/1					4 (3,6)	4
126	126,078ecdc	V/Btb+	2/2	1/1	1/1				4 (3,6)	4
150	AI-12	0			4/4				4 (3,6)	4
045	045, 598, PR4455, 413	V/Btb+	1/1		3/2	1/1		1/1	6 (5,4)	3
001	001, 001ecdc	0				2/2	2/1		4 (3,6)	3
005	005	0	1/1		1/1	1/1			3	3
(CE)013	AI-9-1	0	1/1					2/2	3	3
103	AI-82/1	0	1/1			1/1		1/1	3	3
(CE)288	660	XIb/Btb+	1/1	1/1				1/1	3	3
081	081	0	1/1		2/2				3	2
015	AI-8/0	0	1/1		1/1				2	2
027	027	IIIb/Btb+	1/1	1/1					2	2
029	029	0	1/1			1/1			2	2
(CE)050	050, AI-84	0	1/1		1/1				2	2
056	056	XII			1/1		1/1		2	2
SLO 024	652	V/Btb+					2/1		2	1
003	003	0						1/1	1	1
011/049	049/1	0			1/1				1	1
017	017	VIII						1/1	1	1
018	018	0				1/1			1	1
023	023	IV/Btb+				1/1			1	1
(CE)032	205	tox-						1/1	1	1
(CE)039	039/2	tox-					1/1		1	1
(CE)084	548	tox-						1/1	1	1
(CE)097	AI-60	0					1/1		1	1
127	651	VI/Btb+			1/1				1	1
258	446	XII						1/1	1	1
(CE)342	610	0	1/1						1	1
(CE)365	434	0	1/1						1	1
(CE)448	653	VI/Btb+			1/1				1	1
(CE)602	212	0	1/1						1	1
SLO 133	AI-15	XII			1/1				1	1
SLO 166	661	I						1/1	1	1
All		na	31/7	7/4	31/10	11/2	15/4	17/3	112	12***
Nr. of ribotypes per species			20	6	16	10	9	15		

*Btb + − presence of binary toxin CDT; **including racoons, wild hare, rabbits, goats, partridges, goose, and crow; ***12 countries participated; tox- -nontoxigenic strain (lacking the PaLoc and genes coding for binary toxin CDT); A “CE” prefix, eg. (CE)039, indicates that the PCR ribotype assigment was made in reference laboratory (CDRN Leeds) using the newer capillary electrophoresis-based approach. PCR-ribotypes in the table are ordered according to the number of countries in which the strains were found and then according to the number of strains belonging to that ribotype.

The majority of isolates were from pigs (n = 31; 27.7%) and cattle (n = 31; 27.7%), followed by poultry (n = 11; 9.8%), dogs (n = 10; 8.9%), horses, rabbits (7 isolates each; 6.3%), cats (n = 5; 4.5%), goats (n = 3; 2.7%), partridges and raccoons (2 isolates each; 1.8%), wild hare, crow and goose (1 isolate each; 0.9%).

Only 38 isolates were from animals with clinical signs, 56 were from clinically normal animals and for 18 isolates disease status of the animal was not known.

For 40 animals antibiotic use was unknown and further 52 animals had no history of antibiotic exposure. Prior use of antibiotics was reported for isolates from 20 animals. For 12 of them antibiotic was not specified and for the remaining eight antibiotics given to animals were amoxicillin/clavulanic acid, amoxicillin, colistin, enrofloxacin, cefovecin (6 months before sampling), gentamicin, oxytetracycline, and sulfonamide. Animals treated with antibiotics were cattle (8), cat (1), dog (1), horse (1), pig (2) and rabbit (7). Twenty isolates from animals with reported use of antibiotics belonged to 11 different PCR ribotypes (002, 012, 014/020, 027, 033, 045, 078, 127, (CE)013, (CE)032 and (CE)084). None of those PCR ribotypes was associated with a specific antibiotic.

### Molecular characterization of strains with two PCR ribotyping approaches

Results of agarose gel-based and sequencer-based PCR ribotyping for 112 *C. difficile* isolates are presented in Table 1 and Additional file 1.

**Figure 1:**
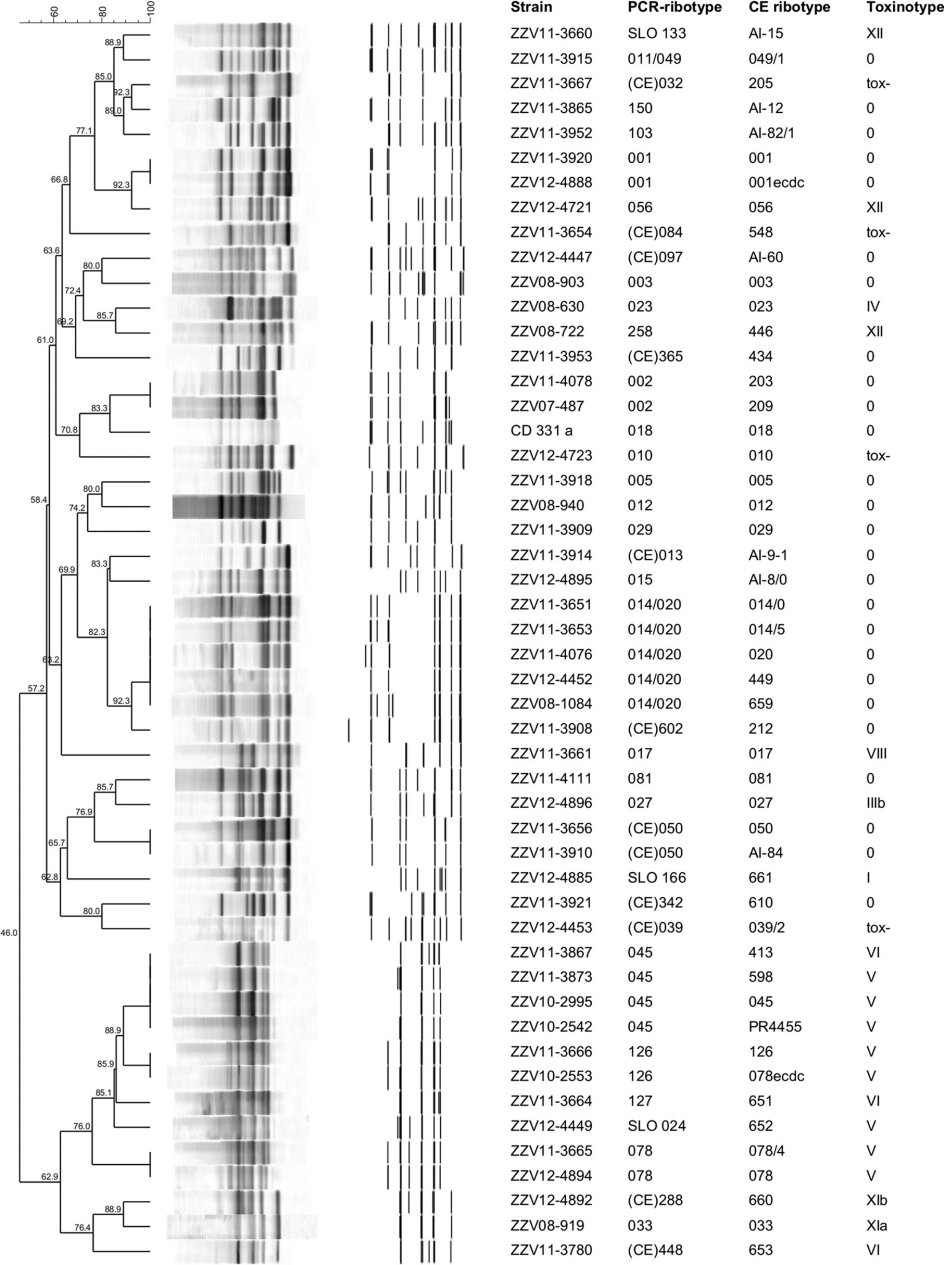
**Dendrogram showing similarities of banding patterns generated with classical agarose gel electrophoresis based PCR ribotyping for all 38 different PCR ribotypes included in the collection.** For each “gel-based” PCR ribotype all profiles generated with capillary electrophoresis PCR ribotyping are added for comparison. A “CE” prefix, eg. (CE) 039, indicates that the ribotype assigment was made in reference laboratory (CDRN Leeds) using the newer capillary electrophoresis-based approach.

Using classical agarose-gel based PCR ribotyping all 112 isolates were distributed into 38 different PCR ribotypes (Table 1). With sequencer-based PCR ribotyping, 50 PCR ribotypes could be identified. This is due to higher discriminatory power of capillary sequencer-based PCR ribotyping for some PCR ribotypes; 001 (001 and 001ecdc), 002 (203 and 209), 014/020 (014/0, 014/5, 020, 449 and 659), (CE)050 (050 and AI-84), 078 (078 and 078/4), 045 (045, 413, 598, PR4455) and 126 (126 and 078ecdc) (Table 1). Comparison of agarose gel PCR ribotypes with the corresponding band profiles generated by capillary electrophoresis-based PCR ribotyping is shown in Figure 1. The discrepancies in the nomenclature between both methods, observed for some of the strains [e.g. PCR ribotype 150 (gel-based) and AI-12 (sequencer-based)], are due to use of a different set of *C. difficile* reference strains and reflects the difficulties in attempts to unify PCR ribotyping nomenclature.

Two most common PCR ribotypes representing 25.9% of all strains were 078 and 014/020. PCR ribotypes with 5 or 6 representatives were 002, 012, 010 (non-toxigenic strain lacking the PaLoc and binary toxin CDT) and 045.

### Molecular characterization of strains by toxinotyping

Within toxigenic isolates (n = 104; 92.9%) 11 different toxinotypes were identified (Table 1). More than a half of the isolates belonged to the nonvariant toxinotype 0 (n = 57; 54.9%). The variant toxinotypes were V (n = 27; 26.0%), XII (n = 4; 3.8%), VI (n = 3; 2.9%), XIa (n = 2; 1.9%), XIb (n = 5; 4.7%), III (n = 2; 1.9%), IV, I, VIII and XIX (n = 1; 1%). Eight strains (7.1%) from four different PCR ribotypes (010, (CE)032, (CE)039, (CE)084) were non-toxigenic.

### Distribution of PCR ribotypes and toxinotypes in different animal hosts and in different countries

**Figure 2:**
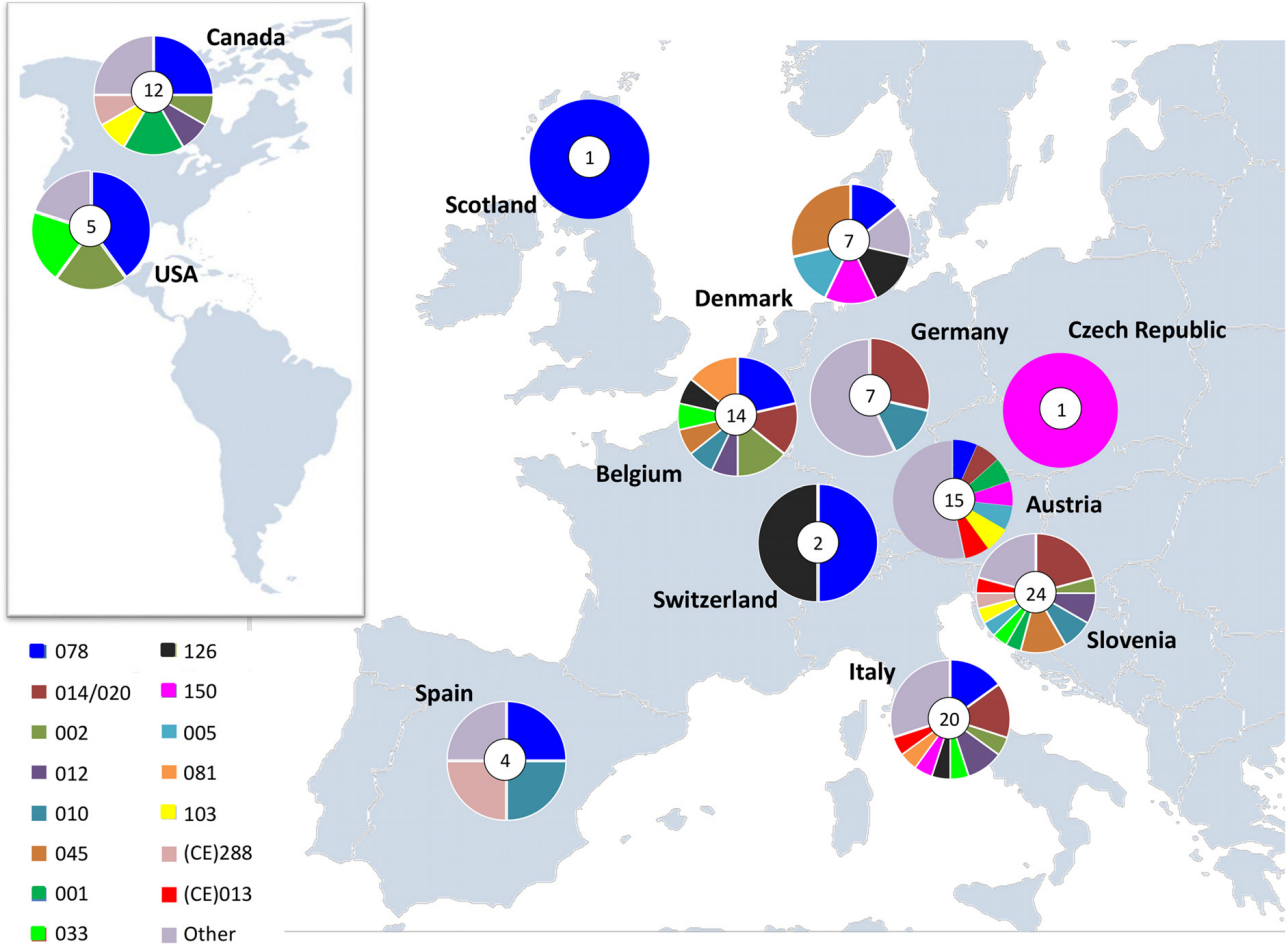
**Geographical distribution of animal associated *C. difficile *PCR ribotypes from participating countries.** Pie charts show proportion of 15 most prevalent PCR ribotypes in the collection for each participating country. The number in the center of pie chart represents the number of isolates from that country. The diversity of strains per country increases with the total number of strains contributed to the collection but also by strains from certain hosts (poultry in case of Slovenia and rabbits in case of Italy).

Table 1 shows distribution of *C. difficile* PCR ribotypes and toxinotypes from collected isolates throughout countries and animal species. Isolates from pigs and cattle were the most frequent and were received from 10 and 7 of the 12 participating countries, respectively. The variability of PCR ribotypes was accordingly also the largest in these two hosts; 20 PCR ribotypes came from cattle and 16 from pigs (Table 1). Distribution of the 15 most prevalent PCR ribotypes in countries is presented in Figure 2.PCR ribotype 014/020 was found in the majority of the animal species included in the collection (pig, cattle, horse, poultry, cat, dog, rabbit and a goat) across 5 different countries (Figure 2). PCR ribotypes that were found only in particular animal species, but in several different countries, were PCR ribotype 150, which was found only in pigs but in four different countries, and PCR ribotype 033, which was found in cattle in three different countries and only in a single case in horse.

Toxinotype V strains of different PCR ribotypes were mainly associated with cattle, horses and pigs and were rarely found in other animals. Also toxinotype XI strains (a and b) were mainly associated with cattle and horses.

## Discussion

Several publications are available about isolation and characterization of *C. difficile* from animals, but they are usually limited to a specific geographical region and data of the different studies are sometimes difficult to compare due to nomenclature of PCR ribotypes that is not unified. The advantage of the international animal *C. difficile* strain collection is in performance of strain typing in the single setting, hereby minimizing the ambiguities in PCR ribotype designations. Additionally, two PCR ribotyping approaches have been used; the standard one using ‘Cardiff’ nomenclature and WEBRIBO based analysis (giving inter-laboratory comparable results independent of Cardiff/Leeds reference strains). This is not a prevalence study with collection of samples from defined number of farms or hosts as was done for hospitals and human isolates in Europe [20]. The isolates in this study were collected from different labs in different countries and the criterion was one PCR ribotype per animal species per lab. Therefore the collected strains are not reflecting the prevalence of PCR ribotypes but are giving a good basis to assess the diversity of animal associated *C. difficile*.

Our results show that variability of PCR ribotypes present in different animal species is large. Isolates from pigs and cattle were most common, perhaps reflecting the importance of *C. difficile* in pigs and concerns about zoonotic transmission from cattle (or more specifically, meat) (Table 1, Table 2). In contrast to cattle and pigs, are cats, dogs, rabbits and poultry clearly understudied and are probably associated with a broader variety of *C. difficile* PCR ribotypes than currently recognized. Only two countries have contributed poultry isolates to the collection, but they represent 10 different PCR ribotypes. Strains from captive rabbits were contributed only by one country, but represent 7 different PCR ribotypes (Additional file 1).

**Table 2:** **Comparison of *C. difficile *** **PCR ribotypes detected in six animal species in different geographical regions**

		**This study**	**Netherlands**	**Germany**	**Switzerland**	**Australia**	**North America**
**Pigs**	Number of ribotypes (reference)	16	3 [10], 1 [7]	20 [15]		1 [18]	7 [21]
	Most prevalent ribotypes	078, 150, 014/020, 045, 002, 081	078	078, 126, 002/2, 126, 413, 049, 598		237	078
**Cattle**	Number of ribotypes (reference)	20	2 [10]	17 [22]	5 [23]	21 [24]	7 [25] 3 [26]
	Most prevalent ribotypes	078, 014/020, 033, 002, 012, 126	012	033, 078, 045, 126	033, 003, 066, 070, 137	127, 033, 126, 056, 087	078, 017, 027, 014, 033
**Cats and dogs**	Number of ribotypes (reference)	9	9 [10]	5 [27]			
	Most prevalent ribotypes	014/020, 010, 001	010, 014, 039, 012	010, 014/020, 039, 045			
**Goats and sheep**	Number of ribotypes (reference)	3			2 [23]	7 [28]	
	Most prevalent ribotypes	010, 014/020, 045			001, 066	101, 137	

Some PCR ribotypes seem to be more often associated with a particular animal host. Many publications report PCR ribotype 078 in pigs [7,10,15] and type 033 in cattle [22,24] (Table 2). Results presented here confirm this observation (Figure 2), but also suggest that the well-known animal-associated PCR ribotype 078 may not be currently present in animals in all countries. In addition to types 078 and 033 some other PCR ribotypes are typically associated with pigs, such as PCR ribotypes 150, 002, 045 and 081 (Table 1). Recent reports from Australia describe a new genotype in terms of PCR ribotype (237) and toxin genes (*tcd*B+, *tcd*A-, *cdt*A + and *cdt*B+) in pigs [18]. PCR ribotype 027 that was prevalent in humans in many countries within the past ten years was in this collection of animal strains found only in USA and Canada (Additional file 1).

Toxinotype V and XI and binary toxin positive strains were initially reported to be the prevalent population of strains isolated from animals (70-100%) [19,29]. However, the results of this study show that nonvariant strains of toxinotype 0 are widespread in animals and that the proportion of binary toxin positive strains can be as low as 35.7%.

All isolates in the collection were distributed into 38 PCR ribotypes, but only a few of those contained five or more isolates, and the majority was represented only by a few or a single isolate (Table 1). This resembles the situation with human strain collections with many different PCR ribotypes, but only a few of them having a large number of isolates [20]. However, as the inclusion criterion for the collection was one PCR ribotype per animal species per laboratory, the number of isolates of a given PCR ribotype does not reflect the actual prevalence of this PCR ribotype in animal host. But the high number of isolates of a given PCR ribotype in the collection does reflect its broader geographic presence and possibly broader spectrum of animal species from which it was isolated.

All animal-associated PCR ribotypes with four or more isolates reported here (Table 1) are among the most prevalent in diverse studies of human isolates [8,11,20,30,31]. In particular PCR ribotype 014/020 is currently the most prevalent type isolated in Europe and in some USA studies and is the only type that was present here in the majority of animal hosts (Table 1). Although type 014/020 is not recognized as hypervirulent and is not associated with outbreaks or severe disease in humans, its ability to colonize many diverse hosts and its ubiquitous presence indicates considerable endemic potential of this particular PCR ribotype [8].

## Conclusions

PCR ribotype 078 is the most prevalent *C. difficile* type associated with this collection of animal isolates, but only with some hosts and in some countries. The second most prevalent PCR ribotype is 014/020 which, in contrast, has broader range of animal hosts. Variability of animal associated PCR ribotypes is substantial and is likely to increase with the number of typed animal isolates. Large overlap of animal associated *C. difficile* PCR ribotypes with human strains furthers concerns about interspecies, including zoonotic, transmission of this important pathogen. Moreover, strains that are prevalent in humans are also prevalent in different animals from different geographic areas, emphasizing that certain strains have a large potential for global dissemination.

## Methods

### Inclusion criteria and requested isolate information

Laboratories from different countries with publications on *C. difficile* in animals were invited to contribute the isolates. Participating laboratories were asked to provide only a single representative PCR ribotype per animal species and laboratory/country. No formal structure was used to guide isolate submission.

Participating laboratories were asked to provide additional information about the individual isolate and the animal host: date and country/city of *C. difficile* isolation or specimen collection, molecular characterization (i.e. PCR ribotype, toxin genes), animal species, age, status (farm, domestic or wild animal), clinical signs and antimicrobial exposure history (when available).

### Cultivation and storage of *C. difficile* isolates

Isolates were first inoculated onto selective medium (CLO, bioMerieux) and subcultured on blood agar plates (COH; bioMerieux). Species identification was confirmed by PCR amplification of *C. difficile* specific gene *cdd* using primers Tim 6 (5′-TCCAATATAATAAATTAGCATTCCA) and Struppi 6 (5′-GGCTATTACACGTAATCCAGATA) [32].

All isolates were stored in Microbank cryogenic vials (Pro-lab Diagnostics) at −80°C.

### PCR ribotyping and toxinotyping

All isolates were characterized by toxinotyping, agarose gel-based PCR ribotyping and capillary gel electrophoresis-based PCR ribotyping.

Agarose gel-based PCR ribotyping was performed as described elsewhere [33]. PCR ribotypes were determined by comparison of banding patterns with the internal library using the BioNumerics software v7.1 (Applied Maths, Belgium). Banding patterns were compared with a reference library of 48 Cardiff type strains. Strains that were not consistent with those in the library were sent to reference laboratory (CDRN Leeds) for confirmation and are named with prefix (CE), indicating that the assignment was made with the newer capillary electrophoresis-based approach. Three strains generated new ribotyping profiles (not previously recognized in Leeds/Cardiff collection) and are designated with the internal nomenclature (SLO and 3-digit code).

Dendrograms were constructed using the Dice coefficient and the unweighted pair group method with arithmetic means (UPGMA). Position tolerance and optimization were set to 1.1%.

Capillary gel electrophoresis-based PCR ribotyping was performed as described previously [34]. Primers described by Bidet and colleagues [35] were used for amplification of intergenic spacer regions (ISRs), with forward primer labelled with a WellRED dye D4-PA (Sigma-Aldrich, Germany). PCR products were analyzed with CEQ™ 8000 Genetic Analysis System (Beckman Coulter) using a 33 cm capillary and gel LPAI. CEQ 600-bp DNA size standard (Beckman-Coulter) was used to determine fragment lengths. Capillary separation conditions were as follows: samples injection voltage of 2 kV over 60 s, separation voltage of 4.8 kV with a total running time of 75 min and a capillary temperature of 50°C.

PCR ribotypes were determined with WEBRIBO database (https://webribo.ages.at/) [34]. Fragment sizes were also imported into BioNumerics software for comparison of banding patterns generated with classical agarose gel electrophoresis and sequencer-based capillary gel electrophoresis.

Toxinotyping was performed as described previously [36]; http://www.mf.uni-mb.si/mikro/tox). Non-toxigenic strains (without the PaLoc, region encoding toxins A and B) were confirmed by amplification of 115 bp long insert with primer pair Lok1/Lok3 [32]. Presence of binary toxin gene *cdt*B was detected as described previously [37].

## Competing interests

All authors declare that they have no competing interests.

## Authors’ contributions

SJ, VZ and MR have designed the study, SJ and VZ have performed typing, MHW has contributed toward typing, all authors have contributed strains and participated at writing. All authors have read and approved the final manuscript.

## Supplementary Material

Additional file 1 **Overview of *C. difficile *** **strains, animal hosts and countries represented in the collection.** Click here for file
