# Supplementary material for: International Clostridium difficile animal strain collection and large diversity of animal associated strains
Source: BMC Microbiol. 2014 Jun 28;14:173. doi: 10.1186/1471-2180-14-173 (PMC4100527; doi:10.1186/1471-2180-14-173)
Supplement: Additional file 1 — Overview of C. difficile strains, animal hosts and countries represented in the collection. [file 1471-2180-14-173-S1.pdf]

Supplementary Table 1. Overview of *C. difficile* strains, animal hosts and countries represented in the collection.

Animal / Country	Cat	Cattle	Dog	Goat	Horse	Pig	Poultry	Rabbit	Goose<br>Wild hare<br>Partridge<br>Crow<br>Racoon	Nr. of strains/ PCR ribotypes
**Austria**		005/0 (005)<br>014/020/0 (014/0)<br>015/0 (AI-8/0)<br>029/0 (029)<br>078/V (078)<br>103/0 (AI-82/1)<br>(CE)050/0 (AI-84)<br>(CE)013/0 (AI-9-1)<br>(CE)602/0 (212)<br>(CE)342/0 (610)<br>(CE)365/0 (434)				011/049/0 (049/1)<br>056/XII (056)<br>150/0 (AI-12)	001/0 (001)			**15/15**
**Belgium**		002/0 (203)<br>010/tox- (010)<br>012/0 (012)<br>014/020/0 (014/0)<br>033/XIb (033)<br>045/V (PR4455)<br>078/V (078)<br>078/V (078)<br>081/0 (081)<br>126/V (078ecdc)			014/020/0 (020)	002/0 (203)<br>078/V (078)<br>081/0 (081)				**14/9**
**Canada**	001/0 (001ecdc)	012/0 (012)<br>078/V (078)	001/0 (001ecdc)<br>002/0 (203)		027/IIIb (027)<br>078/V (078)<br>(CE)288/XIb (660)	015/0 (AI-8/0)<br>078/V (078)			Racoon:<br>103/0 (AI-82/1)<br>SLO 166/I (661)	**12/9**
**Czech Republic**						150/0 /AI-12				**1/1**
**Denmark**						005/0 (005)<br>045/V (598)<br>045/VI (413)<br>078/V (078)<br>126/V (126)<br>150/0 (AI-12)<br>(CE)448/VI (653)				**7/6**
**Germany**	014/020/0 (449)<br>(CE)039/tox-<br>(039/2)<br>SLO 024/V (652)		010/tox- (010)<br>014/020/0 (014/0)<br>SLO 024/V (652)<br>(CE)097/0 (AI-60)							**7/5**

Animal / Country	Cat	Cattle	Dog	Goat	Horse	Pig	Poultry	Rabbit	Goose<br>Wild hare<br>Partridge<br>Crow<br>Racoon	Nr. of strains/ PCR ribotypes
**Italy**		033/XIb (033)<br>126/V (126)				012/XIX (012)<br>014/020/0 (014/0)<br>014/020/0 (014/5)<br>078/V (078)<br>078/V (078/4)<br>081/0 (081)<br>150/0 (AI-12)<br>127/VI (651)<br>(CE)050/0 (050)<br>SLO 133/XII (AI-15)		002/0 (203)<br>012/0 (012)<br>014/020/0 (014/0)<br>078/V (078)<br>(CE)032/tox- (205)<br>(CE)013/0 (AI-9-1)<br>(CE)084/tox- (548)	017/VIII (017)<br>Wild Hare	**20/15**
**Scotland**						078/V (078)				**1/1**
**Slovenia**	014/020/0 (014/0)	002/0 (209)<br>014/020/0 (014/0)	012/0 (012)<br>014/020/0 (659)	010/tox- (010)<br>014/020/0 (014/0)<br>045/V (045)	033/XIa (033)	045/V (045)	001/0 (001)<br>005/0 (005)<br>010/tox- (010)<br>012/0 (012)<br>014/020/0 (014/0)<br>018/0 (018)<br>023/IV (023)<br>029/0 (029)<br>045/V (045)<br>103/0 (AI-82/1)		Crow:<br>003/0 (003)<br><br>Goose:<br>(CE)013/0 (AI-9-1)<br><br>Partridge:<br>(CE)288/XIb (660)<br>258/XII (446)	**24/16**
**Spain**		(CE)288/XIb (660)	010/tox- (010)<br>056/XII (056)			078/V (078)				**4/4**
**Switzerland**					078/V (078)<br>126/V (126)					**2/2**
**USA**		027/IIIb (027)<br>033/XIa (033)<br>078/V (078)				002/0 (203)<br>078/V (078)				**5/4**
**Number of strains (PCR ribotypes) per animal host**	**5<br>(4/5)**	**31<br>(20/22)**	**10<br>(8/9)**	**3<br>(3/3)**	**7<br>(6/6)**	**31<br>(16/20)**	**11<br>(10/10)**	**7<br>(7/7)**	**Na<br>(7/7)**	**Σ112<br>(38/50)**
